# Heat Shock Factor 1 Inhibition: A Novel Anti-Cancer Strategy with Promise for Precision Oncology

**DOI:** 10.3390/cancers15215167

**Published:** 2023-10-27

**Authors:** Khanisyah Erza Gumilar, Yeh Chin, Ibrahim Haruna Ibrahim, Brahmana A. Tjokroprawiro, Jer-Yen Yang, Ming Zhou, Natalie R. Gassman, Ming Tan

**Affiliations:** 1 Graduate Institute of Biomedical Science, China Medical University, Taichung 40402, Taiwanu107003521@cmu.edu.tw (Y.C.); u110210101@cmu.edu.tw (I.H.I.); jyyang@cmu.edu.tw (J.-Y.Y.); 2 Department of Obstetrics and Gynecology, Faculty of Medicine, Airlangga University, Surabaya 60286, Indonesia; brahmanaaskandar@fk.unair.ac.id; 3 Cancer Research Institute, School of Basic Medical Sciences, Central South University, Changsha 410013, China; zhouming2013@csu.edu.cn; 4 Department of Pharmacology and Toxicology, Heersink School of Medicine, The University of Alabama at Birmingham, Birmingham, AL 35294, USA; nrg2@uab.edu; 5 Institute of Biochemistry and Molecular Biology, Center for Cancer Biology, China Medical University, Taichung 406040, Taiwan

**Keywords:** HSF1, heat shock response, cellular stress, cancer stem cells, tumor microenvironment

## Abstract

**Simple Summary:**

Heat shock factor 1 (HSF1) is a transcription factor crucial for cellular stress responses. HSF1 activates heat shock proteins (HSPs) in response to proteotoxic stress, aiding in protein folding and maintaining proteostasis. HSF1 is often overexpressed in various cancer cells, fueling malignancy and indicating a poor prognosis. The mechanisms behind HSF1-induced tumorigenesis are complex and cancer type-dependent. Targeting HSF1 presents a novel cancer treatment strategy.

**Abstract:**

Heat shock factor 1 (HSF1) is a transcription factor crucial for regulating heat shock response (HSR), one of the significant cellular protective mechanisms. When cells are exposed to proteotoxic stress, HSF1 induces the expression of heat shock proteins (HSPs) to act as chaperones, correcting the protein-folding process and maintaining proteostasis. In addition to its role in HSR, HSF1 is overexpressed in multiple cancer cells, where its activation promotes malignancy and leads to poor prognosis. The mechanisms of HSF1-induced tumorigenesis are complex and involve diverse signaling pathways, dependent on cancer type. With its important roles in tumorigenesis and tumor progression, targeting HSF1 offers a novel cancer treatment strategy. In this article, we examine the basic function of HSF1 and its regulatory mechanisms, focus on the mechanisms involved in HSF1′s roles in different cancer types, and examine current HSF1 inhibitors as novel therapeutics to treat cancers.

## 1. Introduction

The transcription factor heat shock factor 1 (HSF1) is crucial in regulating the heat shock response (HSR). HSR is a primary protective mechanism responding to stressful conditions such as elevated temperatures, oxidative stress, heavy metals, and proteotoxic insults [1,2]. When cells are exposed to proteotoxic stress, HSF1 induces the expression of heat shock proteins (HSPs) to act as chaperones, correcting the protein-folding process and maintaining proteostasis [2,3]. Beyond HSR, numerous studies demonstrate that HSF1 orchestrates transcriptional programs distinct from HSR and impacts cell proliferation, survival, and metabolism related to cancer [4,5,6]. HSF1 is overexpressed in multiple cancer types, and its activation supports malignancy and leads to poor prognosis [7,8,9]. As a result, HSF1 is a potential biomarker for identifying the malignancy of cells [10]. The mechanisms of HSF1-induced tumorigenesis are complex and involve diverse pathways, depending on the cancer type. Given the essential role of HSF1 in cancer, researchers are discovering the functions of HSF1 in tumorigenesis and developing HSF1 inhibitors as part of innovative targeted therapy [11].

HSF1 plays an important role in the progression of various cancer types, including those of the breast, lung, ovary, endometrium, and prostate and many other cancers. HSF1 has been reported to control critical oncogenic pathways, influencing cell cycle progression, apoptosis, and angiogenesis. Moreover, its potential to impact immunological responses, modulate the tumor microenvironment, and contribute to the development of therapeutic resistance highlights its importance in cancer biology.

This article briefly discusses the essential functions and regulatory mechanisms of HSF1. Due to the diverse malignant manifestations associated with HSF1 in different cancers, we comprehensively review its functions across various cancer types. Furthermore, we discuss the potential of novel therapeutic agents, specifically HSF1 inhibitors, for cancer treatment.

## 2. HSF1 Biology

### 2.1. HSF1 Structure and Function

**Figure 1:**
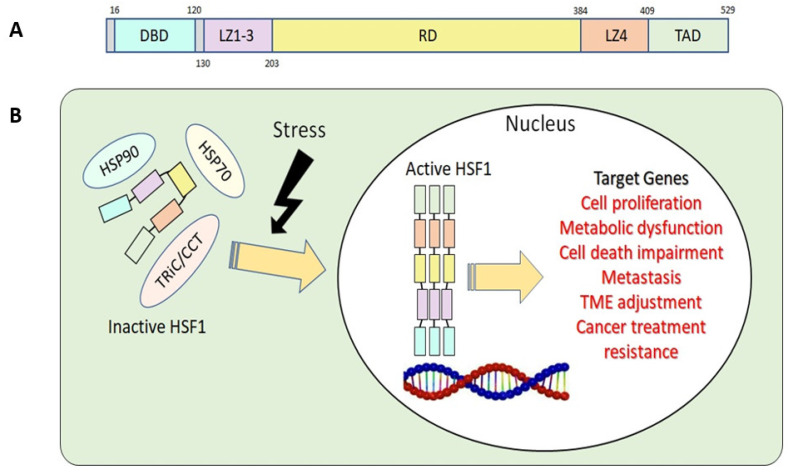
(**A**) Basic structure of human HSF1. DBD, DNA-binding domain; LZ1-3, leucine zipper 1-3; RD, regulatory domain; LZ4, leucine zipper 4; TAD, transactivation domain. (**B**) HSF1 activation related to tumorigenesis.

The structure of the HSF1 protein can be divided into five parts according to their functions: the DNA-binding domain (DBD), leucine zipper 1-3 (LZ1-3), the regulatory domain (RD), leucine zipper 4 (LZ4), and the transactivation domain (TAD) (Figure 1A) [12,13]. Under stress conditions, the N-terminal DBD binds to the target genes’ heat shock element (HSE) during HSR. This binding process requires HSF1 homotrimer formation and subsequent activation [14]. To avoid the continuous activation of HSF1, LZ1-3 and LZ4 form intramolecular interactions to keep HSF1 in its monomeric form and inactive [15]. RD, the domain between LZ1-3 and LZ4, provides an alternative way to regulate HSF1 positively or negatively via modification of specific amino acid residues, known as post-translational modification (PTM) [12,15,16]. Lastly, the C-terminal TAD is related to cell survival once cells undergo heat shock [17].

HSF1 binds to the HSE and functions as a critical regulator of HSR, triggering the transcription of genes encoding HSPs to prevent further damage by protein misfolding and aggregating [1,18]. However, recent studies discovered that, in addition to cytoprotective properties, continuous activation of HSF1 increases cell proliferation and survival and reprograms cell metabolism, similar to cancer cells [4,5,6]. Therefore, research focused on HSF1-induced tumorigenesis has increased dramatically.

### 2.2. HSF1 Regulatory Mechanisms in Normal and Cancer Cells

HSF1 undergoes regulation through various mechanisms, such as intrinsic regulation by LZ1-3 and LZ4 and interactions with chaperones/chaperonins. Additionally, it is subject to various post-transcriptional and post-translational regulatory processes. [12,15]. Furthermore, a recent study also indicated that HSF1 is regulated by non-coding RNA [19].

Upon activation of HSF1 due to stress, it assembles into homotrimers, moves from the cytosol to the nucleus, initiates HSP transcription, and activates HSR. In the nucleus, HSF1 binds to various target genes and activates their respective functions and roles (Figure 1B). As chaperones accumulate in response to HSF1 activation, they engage with HSF1, holding it in the cytosol as a monomer. This interaction attenuates HSRs by rendering HSF1 inactive [12]. This negative-feedback pathway prevents HSF1 overactivation [20].

The regulatory mechanism of HSF1 hinges on post-translational modifications, a process wherein changes occur to the protein after it has been synthesized, for example, phosphorylation [21,22,23,24,25], acetylation [26,27], and SUMOylation [28]. These modifications play a pivotal role in fine-tuning HSF1 activity, influencing its ability to form functional complexes, translocate to the nucleus, and trigger downstream cellular responses.

HSF1’s regulatory mechanisms can be changed in cancer cells, resulting in different patterns of activation and function. Even under non-stress settings, many cancer cells have a heightened and constitutive activation of HSF1. This persistent activation in cancer cells promotes survival and proliferation by increasing the expression of chaperone proteins and anti-apoptotic molecules, which aid in managing proteotoxic stress within rapidly growing malignant cells. Furthermore, HSF1 in cancer cells may be impacted by numerous signaling pathways and oncogenic alterations, resulting in an environment in which HSF1 promotes cancer cell survival and expansion. Further, HSF1 involves multiple layers of cellular regulations to promote malignant phenotypes in cancer cells, including increasing glycolysis to overcome therapy resistance via lactate dehydrogenase A (LDH-A) [29] and promoting autophagy to enhance cell survival via autophagy-related protein 7 (ATG7) [30]. Therefore, the tumorigenic regulation of HSF1 is complicated. Due to its complex mechanism, HSF1 plays different roles in various cancer types.

## 3. HSF1 Involvement in Various Cancer Types

**Figure 2:**
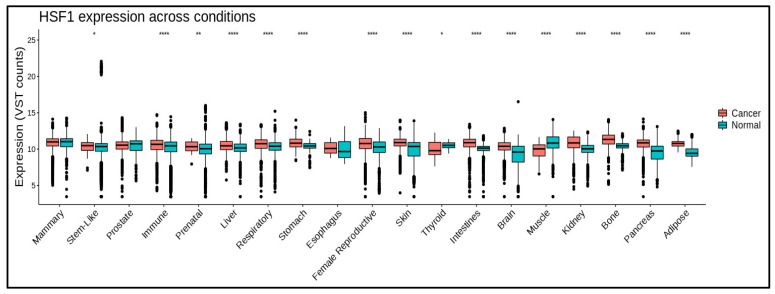
HSF1 mRNA levels in different cancers and normal tissues. HSF1 expression is significantly higher in some cancerous conditions. * means *p* ≤ 0.05; ** means *p* ≤ 0.01; **** means *p* ≤ 0.0001. Data are processed using https://gccri.bishop-lab.uthscsa.edu/correlation-analyzer/ (accessed on 10 October 2023).

HSF1 promotes tumor progression and survival via a variety of methods. It controls gene expression during the cell cycle, apoptosis inhibition, tumor microenvironment modification, angiogenesis, and metastasis. HSF1 expression (Figure 2) is associated with poor prognosis and treatment resistance in various malignancies, including breast, prostate, lung, and ovarian cancers (Table 1). Some studies also suggest that HSF1 expression in tumor tissue also increases significantly according to clinical stage [31,32]. However, in some cancers, there are no significant correlation between HSF1 expression and clinical stage (Figure S1).

HSF1 inhibition has emerged as a possible therapeutic strategy in cancer treatment. HSF1 inhibition has been demonstrated to sensitize cancer cells to chemotherapy, diminish tumor growth, and improve radiation therapy efficacy. Furthermore, HSF1 has emerged as a promising target for cancer therapy.

### 3.1. Breast Cancer

Breast cancer contributes to one out of every four cases of diagnosed cancer and one out of every six cancer deaths. It is the most prevalent cancer in 159 nations and the most common cancer type in the United States [33,34]. HSF1 is a protein that has been investigated extensively for its function in the development of breast cancer.

In breast cancer cells, HSF1 and ErbB2 (HER2) work together to promote glycolysis, cell migration, and invasion. The ErbB2 (HER2) gene codes a protein of the epidermal growth factor receptor (EGFR) family. This receptor family is involved in the regulation of cell growth and division. ErbB2 is involved in signaling pathways that regulate cell growth, survival, and differentiation in normal, healthy cells [32]. However, in rare circumstances, changes in the ErbB2 gene might result in protein overexpression. This overexpression is seen in a subgroup of breast malignancies and is linked to aggressive tumor behavior [33,34].

ErbB2 stimulates the formation of HSF1 trimers and increases HSF1 protein synthesis. HSF1 binds to the LDH-A promoter, increasing LDH-A mRNA levels and leading to higher lactate production and cell growth [8,29,35]. This axis can be identified as a “reprogramming metabolism pathway” and may be an alternative therapeutic strategy for treating ErbB2-overexpressing breast cancers.

HSF1 was also identified as having a link with estrogen (E2) signaling through estrogen receptor α (ERα). When HSF1 is deficient, the level of ERα decreases, weakening the cancer cell’s response to E2 and reducing cell motility and adhesion. HSF1 and ERα work together to regulate gene expression in response to E2, and HSF1 enhances ERα’s activity [36]. HSF1 also becomes activated when it is phosphorylated at Serine326 in response to E2 [37], which differs from HSR [13]. In clinical settings, HSF1 deficiency may increase the effectiveness of hormonal therapies such as Tamoxifen and Palbociclib [36].

In addition, HSF1 was identified as a predictive target gene of microRNA-615-5p, an angiogenesis and tissue repair gene [38]. In breast cancer tissues, low levels of microRNA-615-5p correlated with high levels of HSF1 compared to normal tissues. Furthermore, microRNA-615-5p enhanced apoptosis and reduced the development of breast cancer by downregulating HSF1 expression [38]. These results suggest that increasing microRNA-615-5p, which is essential for tissue repair and blood vessel growth, could be a viable way to treat breast cancer.

Another study investigated FAM3C activation of HSF1, which promoted the growth and motility of breast cancer cells (Figure S2). When TGF-β is overproduced in breast cells, it activates FAM3C-YY1-HSF1 and a protein kinase AKT, which causes cancer cells to grow and migrate [39]. HSF1 is involved in multiple pathways related to breast cancer, indicating that it could be a potential target for targeted breast cancer treatment.

### 3.2. Lung Cancer

Lung cancer is a deadly disease and the leading cause of cancer death, representing 11.4% of cancers diagnosed and 18% of cancer deaths [33]. Non-small-cell lung cancer (NSCLC) is the most common histological type of all lung cancer cases, with a proportion of more than 80% [40].

The first-line treatment for NSCLC involves epidermal growth factor receptor tyrosine kinase inhibitors (EGFR-TKIs). Unfortunately, reports of resistance to these agents in NSCLC are increasing. This resistance is thought to be related to the activation of HSF1, which presents a potential target for overcoming drug resistance. Using KRIBB11, an HSF1 inhibitor, we found a decrease in HSP70 and HSP 27 and BCL2 expression. The reduction in chaperones and anti-apoptotic proteins caused and illustrated cell death [41].

In cases of lung cancer metastasis to the brain, HSF1 plays a crucial role in supporting the survival and proliferation of metastatic cancer cells. [42,43]. A recent study showed that ABL2 tyrosine kinase regulates the expression of the HSF1 protein and its downstream genes. ABL2 is a protein that belongs to the ABL kinase family. The ABL2 gene encodes a nonreceptor tyrosine kinase, and its role in cancer has been studied, particularly in the context of cell signaling, cell migration, and invasion. Inhibiting ABL2 blocks the activity of HSF1 and its targets, which are essential for cancer cell growth and survival (Figure S3) [44]. These data suggest that ABL2 inhibitors could be a promising therapy for metastatic lung cancer with high levels of HSF1.

### 3.3. Ovarian Cancer

The most common type of ovarian cancer is called epithelial ovarian cancer (EOC), which is divided into two groups based on molecular alterations, clinical behavior, and structure [45]. Type I tumors are slow-growing and less aggressive, while Type II tumors spread quickly and are more aggressive. The most frequent Type II cancer is called high-grade serous ovarian cancer (HGSOC), which is very aggressive and often diagnosed late, leading to more ovarian cancer deaths. An easier way to classify EOCs would be into two groups: HGSOC and non-HGSOC. These two groups are biologically different, with non-HGSOC usually growing slowly and being diagnosed early, while HGSOC is naturally aggressive and diagnosed at later stages.

Early detection of HGSOC has numerous advantages, including treating patients promptly and improving prognosis. In early-stage HGSOC, HSF1 is a relevant biomarker since HSF1 can be detected in the blood by tumor-directed autoantibodies (AAb).

Anti-HSF1 antibody detection contributes to the early detection of ovarian cancer. IgA, one of the AAbs employed in the above-cited study, yielded promising outcomes. The levels of anti-HSF1 IgA were higher in the early stages of HGSOC than in the advanced stages [46].

In addition, HSF1 can be employed as a therapy response parameter. Continuing from the previous work [46], the role of IgA in detecting HSI-PO4 contributes to an understanding of treatment response in HGSOC. After a course of combination platinum- and taxol-based treatment (carboplatin and paclitaxel), IgA levels in response to HSF1-PO4 increased considerably, establishing HSF1-PO4 as a possible tumor-associated antigen [47]. Although the initial treatment response reaches 60–80% in HGSOC, patients eventually become platinum-resistant with relapse [48]. HSF1 causes chemoresistance by enhancing autophagy through transcriptional upregulation of ATG7 to maintain cell survival [30]. The role of HSF1 as a biomarker in providing treatment response prediction should be studied further.

Another study revealed that Dickkopf-3 (DKK3), a protein associated with aggressive ovarian cancer, works with HSF1 to control the behavior of cancer-associated fibroblasts (CAFs), which can promote tumor growth and invasion (Figure S4). DKK3 activates a signaling pathway called WNT and reduces the breakdown of another pathway, YAP/TAZ. This makes CAFs more likely to promote tumor growth. DKK3 plays a vital role in ovarian tumors’ stroma (supportive tissue) by controlling how CAFs behave [49].

### 3.4. Endometrial Cancer

In the United States, around 67,000 cases of endometrial cancer were diagnosed, and more than 12,000 people died from it [34]. Being overweight or having excess belly fat increases the risk of endometrial cancer by increasing the amount of estrogen in the body. Other risk factors include taking estrogen pills after menopause, a history of polycystic ovary syndrome (PCOS), and late menopause [50].

Endometrial cancer survival rates vary depending on the stage of diagnosis. The 5-year survival rate for individuals with uterine-confined tumors exceeds 95% but reduces dramatically after the illness spreads outside the uterus, with rates of 69% for patients with regional metastasis and 17% for distant metastases [34].

High HSF1 expression is linked to poor outcomes and disease progression in endometrial cancer. Compared to measurements of the original tumor and complex hyperplasia, HSF1 protein and mRNA expression rose considerably in metastasis. The findings are also compatible with the Kaplan–Meier plot’s survival rate. High HSF1 expression was shown to be adversely related to survival rate [32]. Increased expression of estrogen receptors is associated with the proliferation of endometrial cancer [51,52,53,54]. However, in another study [32], survival analysis showed that high HSF1 expression in both groups (ER- and ER+) was associated with poor survival.

The involvement of estrogen receptors in breast cancer and endometrial cancer is associated with cancer cell proliferation [55]. Intriguingly, a study in breast cancer mentioned that overexpression of HSF1 in ERα-positive breast cancer is associated with decreased reliance on the ERα-controlled transcription program for cancer growth [56]. This suggests that HSF1 may be more effective in controlling cancer cells than the estrogen receptor pathway. Unfortunately, the role of HSF1 and estrogen receptors in endometrial cancer has yet to be fully elucidated. This is an exciting research opportunity and deserves more investigation.

### 3.5. Prostate Cancer

Prostate cancer is the second most common type of cancer in the United States, with an estimated proportion of death cases of 5.6% of all cancer deaths [34]. The 5-year relative survival is 97.5% [34], and most prostate cancers are adenocarcinomas [57]. Among these cancers, some eventually result in castration-resistant prostate cancer (CRPC) or neuroendocrine prostate cancer (NEPC), with the worst prognosis of prostate cancer being histologic type [58].

In prostate cancer, HSF1 is reported as a robust predictive biomarker with high HSF1 mRNA expression and increased nuclear HSF1 shown in patients with advanced prostate cancer [59]. In addition, patients who exhibit nuclear HSF1 abundance and high Gleason scores tend to have poor disease-specific survival [59].

In CRPC and NEPC, HSF1 expression was also highly amplified, with more accumulation of HSF1 than in adenocarcinoma and benign tumors [31]. Overexpression of HSF1 enhances the development of polyploidy, a common feature in cancer cells, and it can improve tumor progression, inferior outcome, progressive stage, and therapy resistance [11,31].

HSF1 inhibition has been proven to reduce cell proliferation in the treatment of prostate cancer. HSF1 inhibition inhibits the expression and transactivation of the androgen receptor (AR), resulting in cell death [60].

Prostate cancer progression requires AR, which highly depends on HSF1-activated multichaperone complexes such as HSP70 and HSP40. This multichaperone complex is essential in HSF1 stability, ligand binding, nuclear translocation, trimerization, and target gene DNA binding [61,62]. In in vitro experiments, Direct Target HSF1 Inhibitor (DTHIB) treatment of prostate cancer cells dose-dependently inhibited the expression of molecular chaperones HSP70 and HSP40 and led to a reduction in AR and diminished prostate-specific antigen (PSA) expression, a marker for prostate cancer progression [31]. In conclusion, HSF1 could provide a novel prognostic marker for patient risk stratification and could lead to new treatments for disease progression and survival.

**Table 1:** HSF1 expression and functions in different cancer types.

Cancer Type	Effect on Tumorigenesis	Reference
Liver cancer	Promotes cell proliferation, growth, migration, invasion, and survival, as well as kinase function, lipid metabolism, and glycolysis	[63,64,65,66,67]
Breast Cancer	Promotes cell motility, metastasis, and survival as well as receptor and kinase maturation, stemness, drug resistance, DNA repair, and EMT	[35,36,37,38,68,69]
Prostate Cancer	Promotes development of polyploidy, high Gleason score, and cancer re-occurrence. Decreases patient survival.	[31,59]
Lung Cancer	Promotes angiogenesis and Metastasis	[41,70,71]
ESCC	Promotes cell survival and expression of HSPs.	[10]
Colorectal Cancer	Promotes expression of anti-apoptotic proteins, cell growth, and glutaminolysis	[71,72]
Endometrial Cancer	Tumor progression	[32]
Ovarian Cancer	Proliferation	[46,47]
	Tumor progression, cell spreading, ECM remodeling, and cancer invasion	[49,73,74]

## 4. HSF1 in Therapeutic Resistance

While chemotherapy or radiotherapy remains the primary approach for treating various cancer types, the ongoing challenge lies in the development of drug resistance by cancer cells despite notable advancements in treatment. Multiple factors contribute to this resistance, with one of the factors being the overexpression or activation of HSF1.

Various cancer cells exhibit increased levels of HSF1, and this upregulation is linked to resistance against chemotherapy. Activated HSF1 boosts the production of HSPs, shielding cancer cells from the harmful impact of chemotherapy drugs. HSPs play a role in preventing protein misfolding and aggregation, aiding protein folding and breakdown, and inhibiting apoptosis—all contributing factors to the development of resistance to chemotherapy [28,62,75,76,77,78]. Cancer cells are stressed when subjected to chemotherapeutic drugs, which can activate HSF1 and the transcription of HSP70 and HSP90, further contributing to therapeutic resistance [68,79].

In addition to its role in HSP regulation, HSF1 also promotes tumor cell survival and proliferation by activating the expression of genes involved in cell cycle regulation [80,81,82], DNA repair [80,83], and angiogenesis [84,85]. Inhibition of HSF1 has been shown to sensitize cancer cells to chemotherapy and reduce tumor growth. Therefore, targeting HSF1 may provide a promising strategy for overcoming chemotherapy resistance in cancer treatment [69,86,87].

HSF1 is also a crucial factor in the transcriptional activation of multidrug resistance 1 (MDR1), which is involved in chemoresistance. The pivotal role of HSF1 in drug resistance can be demonstrated in the binding of HSF1 to the HSE of MDR1. Transfection of active HSF1 increases MDR1 mRNA and protein levels, stimulating drug efflux and the development of drug resistance [39,46]. On the contrary, HSF1 depletion downregulates the transcription of the MDR1 gene in the cells [88].

Another mechanism of multidrug resistance involves the ATP-binding cassette (ABC) transporters, which pump hydrophobic molecules out of the cell. The increasing efflux of drugs mediated by the ABC transporters is one of the most common mechanisms of drug resistance [89]. HSF1 promotes the activation of ABCB1, an ABC transporter. In melanoma cells with HSF1 overexpression, upregulation of ABCB1 gene transcription was prominent. The expression of the ABCB1 gene was found to be primarily dependent on HSF1 in all tested doxorubicin- and paclitaxel-resistant melanoma cell lines [90].

Another study reports an association between HSF1 and F-box and WD repeat domain-containing protein 7 (FBXW7), an important tumor suppressor for human cancer. FBXW7 targets several critical regulators of proliferation, tumor growth, and apoptosis. Drug-resistant cells show decreased FBXW7 expression, leading to increased HSF1 expression and drug resistance [88,91].

Meanwhile, the involvement of HSF1 in chemoresistance was revealed in a study on ATG7 and NBAT1 [30]. Once ATG7 is activated, it leads to the activation of autophagy and increases chemoresistance. NBAT1, a tumor suppressor gene in lung cancer, also regulated associations between HSF1 and ATG7. Overexpression of NBAT1 markedly decreased the binding levels of HSF1 to ATG7 promoter regions, and NBAT1 knockdown showed the opposite effect in NSCLC [92].

In addition to chemoresistance, HSF1 also plays a role in protecting cancer cells from the effects of radiotherapy by boosting the expression of HSPs [93,94,95]. Overexpression of HSF1 leads to radiotherapy resistance in cancer cells. HSF1 activation leads to the upregulation of genes involved in DNA repair, including Rad51, a protein involved in homologous recombination repair, a critical pathway for repairing DNA double-strand breaks induced by ionizing radiation [83,96,97]. Furthermore, HSF1 has been shown to regulate the expression of several anti-apoptotic genes, including Bcl-2 [98,99], which can protect cancer cells from radiation-induced apoptosis. HSF1 has also been shown to activate the NF-κB pathway [100,101,102], which regulates inflammation and immune responses. These three mechanisms may enhance radioresistance by inducing the expression of several pro-survival genes, including HSPs.

High expressions of HSP27, HSP70, and HSP90 exert a radioresistant effect through the anti-apoptotic signaling pathway. In experiments using radioresistant lung cancer cells, a knockdown of HSF1 and administration of an HSP90 inhibitor resulted in a high level of cell apoptosis and increased cell sensitivity to radiotherapy [103]. Apart from these positive effects, the inhibition of HSP90 induces the release of HSF1 from the HSP90 complex, thereby stimulating the transcription of the cytoprotective chaperones HSP70 and HSP27 [8,42,104]. Therefore, this forms a feedback loop to counteract the effect of HSP90 inhibition. Thus, combining the HSP90 inhibitor with the HSF1 inhibitor may achieve a better therapeutic effect. HSP70 is one of the main proteins in response to hyperthermia, including radiotherapy. Upon cellular stress, HSF1 is activated, followed by the upregulation of HSP70. Key actions of HSP70 mediate anti-apoptotic function, regulate multiple intercellular signaling, and induce inflammatory and anti-inflammatory responses that affect cell survival [105,106].

## 5. Targeting HSF1 for Cancer Treatment

Inhibitors of HSF1 are compounds designed to block or modulate the activity of HSF1. These inhibitors have been studied for their potential therapeutic applications, especially in cancer treatment, where HSF1 is often upregulated and contributes to the survival and growth of cancer cells.

**Figure 3:**
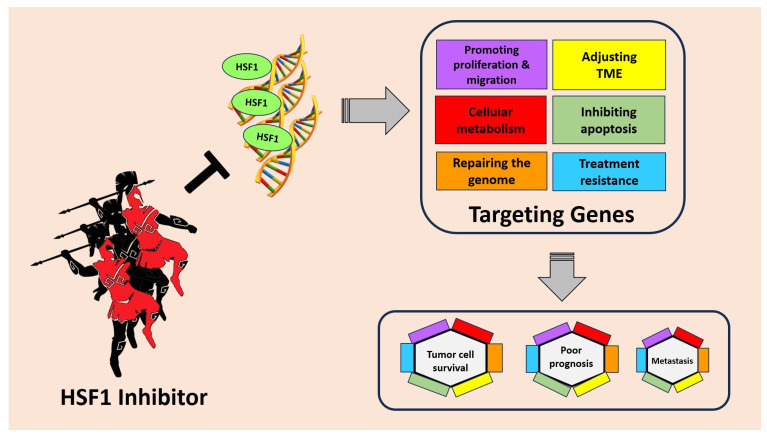
HSF1 plays an important role in tumor cell survival, poor prognosis, and metastasis through several mechanisms. HSF1 can increase the expression of genes involved in anti-apoptotic mechanisms, cellular metabolism, promoting migration, and even drug resistance. By inhibiting HSF1, cancer progression can be suppressed and provide better hope to patients.

Targeting HSF1 for cancer treatment holds great promise as an innovative therapeutic strategy. HSF1, known for its role in cellular stress response, has emerged as a critical regulator in tumorigenesis. In cancer cells, HSF1 becomes hyperactivated, leading to the overexpression of genes involved in anti-apoptotic mechanisms and cellular metabolism. This heightened HSF1 activity also promotes the migration and invasion of cancer cells, facilitating tumor spread to distant sites. Moreover, HSF1 contributes to drug resistance, making cancer treatments less effective (Figure 3).

In light of these findings, scientists have explored inhibiting HSF1 as a potential therapeutic approach (Table 2). By targeting HSF1, researchers aim to disrupt the pro-cancer effects of this transcription factor. Targeting HSF1 as a cancer therapy is currently in the pre-clinical stage, and it is regarded as a promising cancer treatment strategy. In several malignancies, a decrease in HSF1 activity can inhibit aneuploidy and cancer cell proliferation [104].

Despite successfully blocking HSF1 in vitro and in animal models, each agent has therapeutic limitations. Under stress, HSF1 plays a vital role in cancer and normal cells. Inhibiting HSF1 for anti-cancer treatment can be harmful to normal cells. As a result, it is critical to specifically identify and target cancer cells to reduce cytotoxic effects on normal cells. This necessitates the improvement of existing drugs through synthetic techniques that change functional groups/motifs or the identification and isolation of new natural molecules capable of overcoming possible off-target difficulties [107].

Most current inhibitors indirectly interfere with HSF1, lacking specificity and potency. Developing direct small-molecule inhibitors for HSF1 is challenging due to its complex structure. Moreover, the mechanisms of HSF1 in tumorigenesis and development are complicated, involve diverse signaling pathways, and may depend on different cancer types. Recent advancements in HSF1 drug development have brought renewed hope, exemplified by the discovery of direct HSF1 inhibitors such as DTHIB [31,108] and CCT361814 [109,110,111]. These inhibitors have demonstrated potent and specific suppression of tumor growth in pre-clinical animal studies while displaying low toxicity to normal tissues. Encouragingly, CCT361814 has entered Phase I clinical trials. The prospect of developing new generations of HSF1 inhibitors, especially those directly targeting HSF1 itself, holds promise. Additionally, future research should focus on identifying biomarkers for patient selection and monitoring therapeutic effects. With these advancements, a targeted therapy against HSF1 can be developed, providing therapeutic benefits to patients in the near future.

A lack of possible target locations in the tertiary structure makes developing HSF1 inhibitors problematic. HSF-1 is a transcription factor with relatively weak “druggability” [107,112]. Furthermore, its complex activation pathway involves several components, including multichaperone complexes and various PTMs. Nonetheless, promising HSF1 inhibitors have been developed, frequently derived from natural compounds or synthesized chemical structures [87,113]. Below are examples of HSF1 inhibitors tested in vitro and in vivo. So far, NXP800 (CCT361814) is the only HSF1 inhibitor that has entered clinical trials. It is expected that, in the near future, other HSF1 inhibitors will enter clinical trials.

**Table 2:** HSF1 inhibitors.

Agents	Source	Cancer Type	Refs.
Cantharidin	Blister beetles (*Meloidae* spp.)	colon cancer; lung cancer; prostate cancer; breast cancer	[114]
CCT251236	Bisamide	ovarian cancer	[112]
Dorsomorphin		colon cancer; prostate cancer	[115]
I_HSF1_115	Thiazole acrylamide	multiple myeloma; breast cancer	[116,117]
KNK437	Benzylidene lactam	colon cancer; squamous cell carcinoma; breast cancer	[117,118,119]
KRIBB11	Pyridinediamine	multiple myeloma; lung cancer	[41,117,120,121,122]
NZ28	Emetine	myeloma; prostate cancer; lung cancer; breast cancer	[97,123]
NXP800 (CCT361814)	Bisamide	multiple myeloma; solid tumor (under clinical trial)	[109,110,111]
NZ28	Emetine	myeloma; prostate cancer; lung cancer; breast cancer	[97,123]
PW3405	Anthraquinone	HeLa cancer cells	[113,117]
Quercetin	plant pigment (flavonoid)	liver and breast cancer	[124]
Rohinitib (rocaglamide/rocaglates)	Flavaglines;	leukemia	[79,125]
SISU-102 (DTHIB)		prostate cancer; leukemia	[31,108]
SNS-032	Sulfur compounds	leukemia	[126,127,128]
Triptolide	*Tripterygium wilfordii*	chronic lymphocytic leukemia; pancreatic cancer; liver cancer multiple myeloma	[129] [130] [131]
2,4-Bis (4-hydroxy benzyl) phenol	*Gastrodia elata*	lung cancer	[131]
4,6-disubstituted pyrimidines	Aromatic heterocyclic organic compound	osteosarcoma	[128]

## 6. Conclusions

HSF1 inhibition has emerged as a possible cancer therapeutic method. HSF1, a protein involved in cellular stress responses, is frequently overexpressed in cancer cells, contributing to tumor growth and resistance to treatment. HSF1 inhibition has been shown in studies to effectively diminish critical cancer features such as cell proliferation, survival, and metastasis. HSF1 inhibitors interfere with protein folding, reducing HSF1’s ability to bind to DNA, disrupting its involvement in gene activation. Notably, blocking HSF1 has been shown to improve the efficacy of traditional chemotherapy, radiation therapy, and targeted treatments, suggesting its compatibility with these standard therapies.

While laboratory studies have produced encouraging findings, more research is needed to enhance HSF1 inhibitors in terms of efficacy, selectivity, and safety. Scientists are hard at work designing potent drugs that directly target HSF1. This includes determining effective combinations with existing medicines and identifying patient demographics that would benefit the most. Understanding the processes through which cancer cells gain resistance to HSF1 inhibition is also an important line of research. To improve the efficacy of HSF1 inhibitors, researchers are investigating combination techniques combining other targeted medicines.

In conclusion, HSF1 inhibitors have enormous potential as a novel approach to cancer treatment. By focusing on HSF1, researchers hope to overcome treatment resistance, improve the efficacy of existing medicines, and eventually improve outcomes for people with cancer. The current research efforts pave the path for a more refined and all-encompassing approach to cancer therapies.

## Supplementary Materials

The following supporting information can be downloaded at: https://www.mdpi.com/article/10.3390/cancers15215167/s1, Figure S1: The association of HSF1 expression with clinical stages. The analysis shows that lung cancer showed statistically significant differences in HSF1 expression at different stages, but there are no statistically differences in the other three cancer types we analysed; Figure S2: FAM3C-YY1-HSF1 signalling axis in the pathogenesis of breast cancer. FAM3C-YY1-HSF1 signalling axis is essential for TGFβ-promoted proliferation and migration of breast cancer cells; Figure S3: Model diagram illustrating ABL2-HSF1-E2F signaling in lung adenocarcinoma brain metastasis. First, ABL2 interacts with HSF1. HSF1 will oligomerize and accumulate in the nucleus in response to stress by binding to degenerate HSE of E2F target genes. The result of this process is the activation of the G2/M checkpoint which contributes to cell survival and cell proliferation; Figure S4: HSF1 in CAFs upregulates DKK3 which potentiates YAP/TAZ signaling. YAP promotes actomyosin contractility leading to extracellular matrix (ECM) remodeling and cancer cell growth and invasion.

## References

[1] Akerfelt M., Morimoto R.I., Sistonen L. (2010). Heat shock factors: Integrators of cell stress, development and lifespan. Nat. Rev. Mol. Cell Biol..

[2] Morimoto R.I. (2011). The heat shock response: Systems biology of proteotoxic stress in aging and disease. Cold Spring Harb. Symp. Quant. Biol..

[3] Richter K., Haslbeck M., Buchner J. (2010). The heat shock response: Life on the verge of death. Mol. Cell.

[4] Jiang S., Tu K., Fu Q., Schmitt D.C., Zhou L., Lu N., Zhao Y. (2015). Multifaceted roles of HSF1 in cancer. Tumour Biol..

[5] Dai C., Sampson S.B. (2016). HSF1: Guardian of Proteostasis in Cancer. Trends Cell Biol..

[6] Li J., Labbadia J., Morimoto R.I. (2017). Rethinking HSF1 in Stress, Development, and Organismal Health. Trends Cell Biol..

[7] Chen F., Fan Y., Cao P., Liu B., Hou J., Zhang B., Tan K. (2021). Pan-Cancer Analysis of the Prognostic and Immunological Role of HSF1: A Potential Target for Survival and Immunotherapy. Oxid. Med. Cell. Longev..

[8] Wang G., Cao P., Fan Y., Tan K. (2020). Emerging roles of HSF1 in cancer: Cellular and molecular episodes. Biochim. Biophys. Acta Rev. Cancer.

[9] Mendillo M.L., Santagata S., Koeva M., Bell G.W., Hu R., Tamimi R.M., Fraenkel E., Ince T.A., Whitesell L., Lindquist S. (2012). HSF1 Drives a Transcriptional Program Distinct from Heat Shock to Support Highly Malignant Human Cancers. Cell.

[10] Wan T., Shao J., Hu B., Liu G., Luo P., Zhou Y. (2018). Prognostic role of HSF1 overexpression in solid tumors: A pooled analysis of 3,159 patients. Onco Targets Ther..

[11] Dong B., Jaeger A.M., Thiele D.J. (2019). Inhibiting Heat Shock Factor 1 in Cancer: A Unique Therapeutic Opportunity. Trends Pharmacol. Sci..

[12] Gomez-Pastor R., Burchfiel E.T., Thiele D.J. (2018). Regulation of heat shock transcription factors and their roles in physiology and disease. Nat. Rev. Mol. Cell Biol..

[13] Dayalan Naidu S., Dinkova-Kostova A.T. (2017). Regulation of the mammalian heat shock factor 1. FEBS J..

[14] Neudegger T., Verghese J., Hayer-Hartl M., Hartl F.U., Bracher A. (2016). Structure of human heat-shock transcription factor 1 in complex with DNA. Nat. Struct. Mol. Biol..

[15] Anckar J., Sistonen L. (2011). Regulation of HSF1 function in the heat stress response: Implications in aging and disease. Annu. Rev. Biochem..

[16] Kmiecik S.W., Mayer M.P. (2022). Molecular mechanisms of heat shock factor 1 regulation. Trends Biochem. Sci..

[17] Ravarani C.N., Erkina T.Y., De Baets G., Dudman D.C., Erkine A.M., Babu M.M. (2018). High-throughput discovery of functional disordered regions: Investigation of transactivation domains. Mol. Syst. Biol..

[18] Pincus D. (2020). Regulation of Hsf1 and the Heat Shock Response. Adv. Exp. Med. Biol..

[19] Li G., Kryczek I., Nam J., Li X., Li S., Li J., Wei S., Grove S., Vatan L., Zhou J. (2021). LIMIT is an immunogenic lncRNA in cancer immunity and immunotherapy. Nat. Cell Biol..

[20] Masser A.E., Ciccarelli M., Andreasson C. (2020). Hsf1 on a leash—Controlling the heat shock response by chaperone titration. Exp. Cell Res..

[21] Guettouche T., Boellmann F., Lane W.S., Voellmy R. (2005). Analysis of phosphorylation of human heat shock factor 1 in cells experiencing a stress. BMC Biochem..

[22] Holmes B., Benavides-Serrato A., Freeman R.S., Landon K.A., Bashir T., Nishimura R.N., Gera J. (2018). mTORC2/AKT/HSF1/HuR constitute a feed-forward loop regulating Rictor expression and tumor growth in glioblastoma. Oncogene.

[23] Srivastava P., Takii R., Okada M., Fujimoto M., Nakai A. (2021). MED12 interacts with the heat-shock transcription factor HSF1 and recruits CDK8 to promote the heat-shock response in mammalian cells. FEBS Lett..

[24] Chou S.D., Prince T., Gong J., Calderwood S.K. (2012). mTOR is essential for the proteotoxic stress response, HSF1 activation and heat shock protein synthesis. PLoS ONE.

[25] Huang C.Y., Lee F.L., Peng S.F., Lin K.H., Chen R.J., Ho T.J., Tsai F.J., Padma V.V., Kuo W.W., Huang C.Y. (2018). HSF1 phosphorylation by ERK/GSK3 suppresses RNF126 to sustain IGF-IIR expression for hypertension-induced cardiomyocyte hypertrophy. J. Cell. Physiol..

[26] Raychaudhuri S., Loew C., Korner R., Pinkert S., Theis M., Hayer-Hartl M., Buchholz F., Hartl F.U. (2014). Interplay of acetyltransferase EP300 and the proteasome system in regulating heat shock transcription factor 1. Cell.

[27] Westerheide S.D., Anckar J., Stevens S.M., Sistonen L., Morimoto R.I. (2009). Stress-inducible regulation of heat shock factor 1 by the deacetylase SIRT1. Science.

[28] Kmiecik S.W., Le Breton L., Mayer M.P. (2020). Feedback regulation of heat shock factor 1 (Hsf1) activity by Hsp70-mediated trimer unzipping and dissociation from DNA. EMBO J..

[29] Zhao Y.H., Zhou M., Liu H., Ding Y., Khong H.T., Yu D., Fodstad O., Tan M. (2009). Upregulation of lactate dehydrogenase A by ErbB2 through heat shock factor 1 promotes breast cancer cell glycolysis and growth. Oncogene.

[30] Desai S., Liu Z., Yao J., Patel N., Chen J., Wu Y., Ahn E.E., Fodstad O., Tan M. (2013). Heat shock factor 1 (HSF1) controls chemoresistance and autophagy through transcriptional regulation of autophagy-related protein 7 (ATG7). J. Biol. Chem..

[31] Dong B., Jaeger A.M., Hughes P.F., Loiselle D.R., Hauck J.S., Fu Y., Haystead T.A., Huang J., Thiele D.J. (2020). Targeting therapy-resistant prostate cancer via a direct inhibitor of the human heat shock transcription factor 1. Sci. Transl. Med..

[32] Engerud H., Tangen I.L., Berg A., Kusonmano K., Halle M.K., Oyan A.M., Kalland K.H., Stefansson I., Trovik J., Salvesen H.B. (2014). High level of HSF1 associates with aggressive endometrial carcinoma and suggests potential for HSP90 inhibitors. Br. J. Cancer.

[33] Sung H., Ferlay J., Siegel R.L., Laversanne M., Soerjomataram I., Jemal A., Bray F. (2021). Global Cancer Statistics 2020: GLOBOCAN Estimates of Incidence and Mortality Worldwide for 36 Cancers in 185 Countries. CA Cancer J. Clin..

[34] Society A.C. (2021). Cancer Facts & Figures 2021.

[35] He L., Lv S., Ma X., Jiang S., Zhou F., Zhang Y., Yu R., Zhao Y. (2022). ErbB2 promotes breast cancer metastatic potential via HSF1/LDHA axis-mediated glycolysis. Med. Oncol..

[36] Vydra N., Janus P., Kus P., Stokowy T., Mrowiec K., Toma-Jonik A., Krzywon A., Cortez A.J., Wojtas B., Gielniewski B. (2021). Heat shock factor 1 (HSF1) cooperates with estrogen receptor α (ERα) in the regulation of estrogen action in breast cancer cells. eLife.

[37] Vydra N., Janus P., Toma-Jonik A., Stokowy T., Mrowiec K., Korfanty J., Długajczyk A., Wojtaś B., Gielniewski B., Widłak W. (2019). 17β-Estradiol Activates HSF1 via MAPK Signaling in ERα-Positive Breast Cancer Cells. Cancers.

[38] Liu K., Ma R. (2021). MicroRNA-615-5p regulates the proliferation and apoptosis of breast cancer cells by targeting HSF1. Exp. Ther. Med..

[39] Yang W., Feng B., Meng Y., Wang J., Geng B., Cui Q., Zhang H., Yang Y., Yang J. (2019). FAM3C-YY1 axis is essential for TGFbeta-promoted proliferation and migration of human breast cancer MDA-MB-231 cells via the activation of HSF1. J. Cell. Mol. Med..

[40] Sanchez-Ortega M., Carrera A.C., Garrido A. (2021). Role of NRF2 in Lung Cancer. Cells.

[41] Lee S., Jung J., Lee Y.J., Kim S.K., Kim J.A., Kim B.K., Park K.C., Kwon B.M., Han D.C. (2021). Targeting HSF1 as a Therapeutic Strategy for Multiple Mechanisms of EGFR Inhibitor Resistance in EGFR Mutant Non-Small-Cell Lung Cancer. Cancers.

[42] Dai C., Whitesell L., Rogers A.B., Lindquist S. (2007). Heat shock factor 1 is a powerful multifaceted modifier of carcinogenesis. Cell.

[43] Hoj J.P., Mayro B., Pendergast A.M. (2020). The ABL2 kinase regulates an HSF1-dependent transcriptional program required for lung adenocarcinoma brain metastasis. Proc. Natl. Acad. Sci. USA.

[44] Kim J., Park E.Y., Kim O., Schilder J.M., Coffey D.M., Cho C.H., Bast R.C. (2018). Cell Origins of High-Grade Serous Ovarian Cancer. Cancers.

[45] Wilson A.L., Moffitt L.R., Duffield N., Rainczuk A., Jobling T.W., Plebanski M., Stephens A.N. (2018). Autoantibodies against HSF1 and CCDC155 as Biomarkers of Early-Stage, High-Grade Serous Ovarian Cancer. Cancer Epidemiol. Biomark. Prev..

[46] Moody R., Wilson K., Kampan N.C., McNally O.M., Jobling T.W., Jaworowski A., Stephens A.N., Plebanski M. (2021). Mapping Epitopes Recognised by Autoantibodies Shows Potential for the Diagnosis of High-Grade Serous Ovarian Cancer and Monitoring Response to Therapy for This Malignancy. Cancers.

[47] van Zyl B., Tang D., Bowden N.A. (2018). Biomarkers of platinum resistance in ovarian cancer: What can we use to improve treatment. Endocr. Relat. Cancer.

[48] Ferrari N., Ranftl R., Chicherova I., Slaven N.D., Moeendarbary E., Farrugia A.J., Lam M., Semiannikova M., Westergaard M.C.W., Tchou J. (2019). Dickkopf-3 links HSF1 and YAP/TAZ signalling to control aggressive behaviours in cancer-associated fibroblasts. Nat. Commun..

[49] American College of Obstetricians and Gynecologists’ Committee on Practice Bulletins—Gynecology (2018). Polycystic Ovary Syndrome. ACOG Pract. Bull..

[50] Hu G., Zhang J., Zhou X., Liu J., Wang Q., Zhang B. (2020). Roles of estrogen receptor α and β in the regulation of proliferation in endometrial carcinoma. Pathol. Res. Pract..

[51] Rodriguez A.C., Blanchard Z., Maurer K.A., Gertz J. (2019). Estrogen Signaling in Endometrial Cancer: A Key Oncogenic Pathway with Several Open Questions. Horm. Cancer.

[52] Yu K., Huang Z.Y., Xu X.L., Li J., Fu X.W., Deng S.L. (2022). Estrogen Receptor Function: Impact on the Human Endometrium. Front. Endocrinol..

[53] Guha P., Sen K., Chowdhury P., Mukherjee D. (2023). Estrogen receptors as potential therapeutic target in endometrial cancer. J. Recept. Signal Transduct..

[54] Ranhotra H.S. (2018). The estrogen-related receptors in metabolism and cancer: Newer insights. J. Recept. Signal Transduct. Res..

[55] Silveira M.A., Tav C., Berube-Simard F.A., Cuppens T., Leclercq M., Fournier E., Cote M.C., Droit A., Bilodeau S. (2021). Modulating HSF1 levels impacts expression of the estrogen receptor alpha and antiestrogen response. Life Sci. Alliance.

[56] Schatten H. (2018). Brief Overview of Prostate Cancer Statistics, Grading, Diagnosis and Treatment Strategies; Part of the Advances in Experimental Medicine and Biology book series (AEMB, volume 1095). Cell & Molecular Biology of Prostate Cancer.

[57] Watson P.A., Arora V.K., Sawyers C.L. (2015). Emerging mechanisms of resistance to androgen receptor inhibitors in prostate cancer. Nat. Rev. Cancer.

[58] Björk J.K., Ahonen I., Mirtti T., Erickson A., Rannikko A., Bützow A., Nordling S., Lundin J., Lundin M., Sistonen L. (2018). Increased HSF1 expression predicts shorter disease-specific survival of prostate cancer patients following radical prostatectomy. Oncotarget.

[59] Xia Y., Wang M., Beraldi E., Cong M., Zoubeidi A., Gleave M., Peng L. (2015). A Novel Triazole Nucleoside Suppresses Prostate Cancer Cell Growth by Inhibiting Heat Shock Factor 1 and Androgen Receptor. Anti-Cancer Agents Med. Chem..

[60] Wyatt A.W., Gleave M.E. (2015). Targeting the adaptive molecular landscape of castration-resistant prostate cancer. EMBO Mol. Med..

[61] Moses M.A., Kim Y.S., Rivera-Marquez G.M., Oshima N., Watson M.J., Beebe K.E., Wells C., Lee S., Zuehlke A.D., Shao H. (2018). Targeting the Hsp40/Hsp70 Chaperone Axis as a Novel Strategy to Treat Castration-Resistant Prostate Cancer. Cancer Res..

[62] Fang F., Chang R., Yang L. (2012). Heat shock factor 1 promotes invasion and metastasis of hepatocellular carcinoma in vitro and in vivo. Cancer.

[63] Shen Z., Yin L., Zhou H., Ji X., Jiang C., Zhu X., He X. (2021). Combined inhibition of AURKA and HSF1 suppresses proliferation and promotes apoptosis in hepatocellular carcinoma by activating endoplasmic reticulum stress. Cell. Oncol..

[64] Li M., Hu J., Jin R., Cheng H., Chen H., Li L., Guo K. (2020). Effects of LRP1B Regulated by HSF1 on Lipid Metabolism in Hepatocellular Carcinoma. J. Hepatocell. Carcinoma.

[65] Liu H.T., Huang D.A., Li M.M., Liu H.D., Guo K. (2019). HSF1: A mediator in metabolic alteration of hepatocellular carcinoma cells in cross-talking with tumor-associated macrophages. Am. J. Transl. Res..

[66] Zhang N., Wu Y., Lyu X., Li B., Yan X., Xiong H., Li X., Huang G., Zeng Y., Zhang Y. (2017). HSF1 upregulates ATG4B expression and enhances epirubicin-induced protective autophagy in hepatocellular carcinoma cells. Cancer Lett..

[67] Santagata S., Hu R., Lin N.U., Mendillo M.L., Collins L.C., Hankinson S.E., Schnitt S.J., Whitesell L., Tamimi R.M., Lindquist S. (2011). High levels of nuclear heat-shock factor 1 (HSF1) are associated with poor prognosis in breast cancer. Proc. Natl. Acad. Sci. USA.

[68] Yang T., Ren C., Lu C., Qiao P., Han X., Wang L., Wang D., Lv S., Sun Y., Yu Z. (2019). Phosphorylation of HSF1 by PIM2 Induces PD-L1 Expression and Promotes Tumor Growth in Breast Cancer. Cancer Res..

[69] Yun H.H., Baek J.Y., Seo G., Kim Y.S., Ko J.H., Lee J.H. (2018). Effect of BIS depletion on HSF1-dependent transcriptional activation in A549 non-small cell lung cancer cells. Korean J. Physiol. Pharmacol..

[70] Song P., Feng L., Li J., Dai D., Zhu L., Wang C., Li J., Li L., Zhou Q., Shi R. (2020). β-catenin represses miR455-3p to stimulate m6A modification of HSF1 mRNA and promote its translation in colorectal cancer. Mol. Cancer.

[71] Cen H., Zheng S., Fang Y.M., Tang X.P., Dong Q. (2004). Induction of HSF1 expression is associated with sporadic colorectal cancer. World J. Gastroenterol..

[72] Scherz-Shouval R., Santagata S., Mendillo M.L., Sholl L.M., Ben-Aharon I., Beck A.H., Dias-Santagata D., Koeva M., Stemmer S.M., Whitesell L. (2014). The reprogramming of tumor stroma by HSF1 is a potent enabler of malignancy. Cell.

[73] Heng B.C., Zhang X., Aubel D., Bai Y., Li X., Wei Y., Fussenegger M., Deng X. (2021). An overview of signaling pathways regulating YAP/TAZ activity. Cell. Mol. Life Sci..

[74] Kijima T., Prince T.L., Tigue M.L., Yim K.H., Schwartz H., Beebe K., Lee S., Budzynski M.A., Williams H., Trepel J.B. (2018). HSP90 inhibitors disrupt a transient HSP90-HSF1 interaction and identify a noncanonical model of HSP90-mediated HSF1 regulation. Sci. Rep..

[75] Workman P. (2020). Reflections and Outlook on Targeting HSP90, HSP70 and HSF1 in Cancer: A Personal Perspective. Adv. Exp. Med. Biol..

[76] Cyran A.M., Zhitkovich A. (2022). Heat Shock Proteins and HSF1 in Cancer. Front. Oncol..

[77] Chin Y., Gumilar K.E., Li X.G., Tjokroprawiro B.A., Lu C.H., Lu J., Zhou M., Sobol W., Tan M. (2023). Targeting HSF1 for cancer treatment: Mechanisms and inhibitor development. Theranostic.

[78] Santagata S., Mendillo M.L., Tang Y.C., Subramanian A., Perley C.C., Roche S.P., Wong B., Narayan R., Kwon H., Koeva M. (2013). Tight coordination of protein translation and HSF1 activation supports the anabolic malignant state. Science.

[79] Chang Z., Lu M., Park S.M., Park H.K., Kang H.S., Pak Y., Park J.S. (2012). Functional HSF1 requires aromatic-participant interactions in protecting mouse embryonic fibroblasts against apoptosis via G2 cell cycle arrest. Mol. Cells.

[80] Luft J.C., Benjamin I.J., Mestril R., Dix D.J. (2001). Heat shock factor 1-mediated thermotolerance prevents cell death and results in G2/M cell cycle arrest. Cell Stress Chaperones.

[81] Bruce J.L., Chen C., Xie Y., Zhong R., Wang Y.Q., Stevenson M.A., Calderwood S.K. (1999). Activation of heat shock transcription factor 1 to a DNA binding form during the G(1)phase of the cell cycle. Cell Stress Chaperones.

[82] Fujimoto M., Takii R., Takaki E., Katiyar A., Nakato R., Shirahige K., Nakai A. (2017). The HSF1-PARP13-PARP1 complex facilitates DNA repair and promotes mammary tumorigenesis. Nat. Commun..

[83] Shi X., Deng Z., Wang S., Zhao S., Xiao L., Zou J., Li T., Tan S., Tan S., Xiao X. (2021). Increased HSF1 Promotes Infiltration and Metastasis in Cervical Cancer via Enhancing MTDH-VEGF-C Expression. Onco Targets Ther..

[84] Tian X., Zhou N., Yuan J., Lu L., Zhang Q., Wei M., Zou Y., Yuan L. (2020). Heat shock transcription factor 1 regulates exercise-induced myocardial angiogenesis after pressure overload via HIF-1α/VEGF pathway. J. Cell. Mol. Med..

[85] Gabai V.L., Meng L., Kim G., Mills T.A., Benjamin I.J., Sherman M.Y. (2012). Heat shock transcription factor Hsf1 is involved in tumor progression via regulation of hypoxia-inducible factor 1 and RNA-binding protein HuR. Mol. Cell. Biol..

[86] McConnell J.R., Buckton L.K., McAlpine S.R. (2015). Regulating the master regulator: Controlling heat shock factor 1 as a chemotherapy approach. Bioorg. Med. Chem. Lett..

[87] Mun G.I., Choi E., Lee Y., Lee Y.S. (2020). Decreased expression of FBXW7 by ERK1/2 activation in drug-resistant cancer cells confers transcriptional activation of MDR1 by suppression of ubiquitin degradation of HSF1. Cell Death Dis..

[88] Bukowski K., Kciuk M., Kontek R. (2020). Mechanisms of Multidrug Resistance in Cancer Chemotherapy. Int. J. Mol. Sci..

[89] Vydra N., Toma A., Glowala-Kosinska M., Gogler-Piglowska A., Widlak W. (2013). Overexpression of heat shock transcription factor 1 enhances the resistance of melanoma cells to doxorubicin and paclitaxe. BMC Cancer.

[90] Kourtis N., Moubarak R.S., Aranda-Orgilles B., Lui K., Aydin I.T., Trimarchi T., Darvishian F., Salvaggio C., Zhong J., Bhatt K. (2015). FBXW7 modulates cellular stress response and metastatic potential through HSF1 post-translational modification. Nat. Cell Biol..

[91] Zheng T. (2018). Long noncoding RNA NBAT1 inhibits autophagy via suppression of ATG7 in non-small cell lung cancer. Am. J. Cancer Res..

[92] Gabai V.L., Budagova K.R., Sherman M.Y. (2005). Increased expression of the major heat shock protein Hsp72 in human prostate carcinoma cells is dispensable for their viability but confers resistance to a variety of anticancer agents. Oncogene.

[93] Schilling D., Bayer C., Li W., Molls M., Vaupel P., Multhoff G. (2012). Radiosensitization of normoxic and hypoxic h1339 lung tumor cells by heat shock protein 90 inhibition is independent of hypoxia inducible factor-1α. PLoS ONE.

[94] Zaidi S., McLaughlin M., Bhide S.A., Eccles S.A., Workman P., Nutting C.M., Huddart R.A., Harrington K.J. (2012). The HSP90 inhibitor NVP-AUY922 radiosensitizes by abrogation of homologous recombination resulting in mitotic entry with unresolved DNA damage. PLoS ONE.

[95] Li Q., Martinez J.D. (2011). Loss of HSF1 results in defective radiation-induced G(2) arrest and DNA repair. Radiat. Res..

[96] Schilling D., Kuhnel A., Konrad S., Tetzlaff F., Bayer C., Yaglom J., Multhoff G. (2015). Sensitizing tumor cells to radiation by targeting the heat shock response. Cancer Lett..

[97] Jacobs A.T., Marnett L.J. (2009). HSF1-mediated BAG3 expression attenuates apoptosis in 4-hydroxynonenal-treated colon cancer cells via stabilization of anti-apoptotic Bcl-2 proteins. J. Biol. Chem..

[98] Kim H.Y., Kim Y.-S., Yun H.H., Im C.-N., Ko J.-H., Lee J.-H. (2016). ERK-mediated phosphorylation of BIS regulates nuclear translocation of HSF1 under oxidative stress. Exp. Mol. Med..

[99] Janus P., Pakuła-Cis M., Kalinowska-Herok M., Kashchak N., Szołtysek K., Pigłowski W., Widlak W., Kimmel M., Widlak P. (2011). NF-κB signaling pathway is inhibited by heat shock independently of active transcription factor HSF1 and increased levels of inducible heat shock proteins. Genes Cells.

[100] Li W., Hu C., Zhong X., Wu J., Li G. (2022). Melatonin Induces AGS Gastric Cancer Cell Apoptosis via Regulating PERK/eIF2α and HSF1/NF-κB Signaling Pathway. Ann. Clin. Lab. Sci..

[101] Li J., Liu Y., Duan P., Yu R., Gu Z., Li L., Liu Z., Su L. (2018). NF-κB regulates HSF1 and c-Jun activation in heat stress-induced intestinal epithelial cell apoptosis. Mol. Med. Rep..

[102] Kühnel A., Schilling D., Combs S.E., Haller B., Schwab M., Multhoff G. (2019). Radiosensitization of HSF-1 Knockdown Lung Cancer Cells by Low Concentrations of Hsp90 Inhibitor NVP-AUY922. Cells.

[103] Carpenter R.L., Gokmen-Polar Y. (2019). HSF1 as a Cancer Biomarker and Therapeutic Target. Curr. Cancer Drug Targets.

[104] Nytko K.J., Thumser-Henner P., Russo G., Weyland M.S., Rohrer Bley C. (2020). Role of HSP70 in response to (thermo)radiotherapy: Analysis of gene expression in canine osteosarcoma cells by RNA-seq. Sci. Rep..

[105] Multhoff G., Pockley A.G., Schmid T.E., Schilling D. (2015). The role of heat shock protein 70 (Hsp70) in radiation-induced immunomodulation. Cancer Lett..

[106] Velayutham M., Cardounel A.J., Liu Z., Ilangovan G. (2018). Discovering a Reliable Heat-Shock Factor-1 Inhibitor to Treat Human Cancers: Potential Opportunity for Phytochemists. Front. Oncol..

[107] Dong Q., Xiu Y., Wang Y., Hodgson C., Borcherding N., Jordan C., Buchanan J., Taylor E., Wagner B., Leidinger M. (2022). HSF1 is a driver of leukemia stem cell self-renewal in acute myeloid leukemia. Nat. Commun..

[108] Workman P., Clarke P.A., Te Poele R., Powers M., Box G., De Billy E., De Haven Brandon A., Hallsworth A., Hayes A., McCann H. (2022). Discovery and validation of biomarkers to support clinical development of NXP800: A first-in-class orally active, small-molecule HSF1 pathway inhibitor. Eur. J. Cancer.

[109] Menezes K., Aram G., Mirabella F., Johnson D.C., Sherborne A.L., Houlston R.S., Cheeseman M.D., Pasqua E., Clarke P., Workman P. (2017). The Novel Protein HSF1 Stress Pathway Inhibitor Bisamide CCT361814 Demonstrates Pre-Clinical Anti-Tumor Activity in Myeloma. Blood.

[110] Diane Marsolini S.S. A Phase 1 Clinical Study of NXP800 in Subjects With Advanced Cancers. https://clinicaltrials.gov/ct2/show/NCT05226507?term=HSF1&draw=2&rank=3#contacts.

[111] Cheeseman M.D., Chessum N.E., Rye C.S., Pasqua A.E., Tucker M.J., Wilding B., Evans L.E., Lepri S., Richards M., Sharp S.Y. (2017). Discovery of a Chemical Probe Bisamide (CCT251236): An Orally Bioavailable Efficacious Pirin Ligand from a Heat Shock Transcription Factor 1 (HSF1) Phenotypic Screen. J. Med. Chem..

[112] Zhang D., Zhang B. (2016). Selective killing of cancer cells by small molecules targeting heat shock stress response. Biochem. Biophys. Res. Commun..

[113] Kim J.A., Kim Y., Kwon B.M., Han D.C. (2013). The natural compound cantharidin induces cancer cell death through inhibition of heat shock protein 70 (HSP70) and Bcl-2-associated athanogene domain 3 (BAG3) expression by blocking heat shock factor 1 (HSF1) binding to promoters. J. Biol. Chem..

[114] Li N., Wang T., Li Z., Ye X., Deng B., Zhuo S., Yao P., Yang M., Mei H., Chen X. (2019). Dorsomorphin induces cancer cell apoptosis and sensitizes cancer cells to HSP90 and proteasome inhibitors by reducing nuclear heat shock factor 1 levels. Cancer Biol. Med..

[115] Vilaboa N., Bore A., Martin-Saavedra F., Bayford M., Winfield N., Firth-Clark S., Kirton S.B., Voellmy R. (2017). New inhibitor targeting human transcription factor HSF1: Effects on the heat shock response and tumor cell survival. Nucleic Acids Res..

[116] Sharma C., Seo Y.H. (2018). Small Molecule Inhibitors of HSF1-Activated Pathways as Potential Next-Generation Anticancer Therapeutics. Molecules.

[117] Whitesell L., Lindquist S. (2009). Inhibiting the transcription factor HSF1 as an anticancer strategy. Expert Opin. Ther. Targets.

[118] Oommen D., Prise K.M. (2012). KNK437, abrogates hypoxia-induced radioresistance by dual targeting of the AKT and HIF-1α survival pathways. Biochem. Biophys. Res. Commun..

[119] Yoon Y.J., Kim J.A., Shin K.D., Shin D.S., Han Y.M., Lee Y.J., Lee J.S., Kwon B.M., Han D.C. (2011). KRIBB11 inhibits HSP70 synthesis through inhibition of heat shock factor 1 function by impairing the recruitment of positive transcription elongation factor b to the hsp70 promoter. J. Biol. Chem..

[120] Kang M.J., Yun H.H., Lee J.H. (2017). KRIBB11 accelerates Mcl-1 degradation through an HSF1-independent, Mule-dependent pathway in A549 non-small cell lung cancer cells. Biochem. Biophys. Res. Commun..

[121] Fok J.H.L., Hedayat S., Zhang L., Aronson L.I., Mirabella F., Pawlyn C., Bright M.D., Wardell C.P., Keats J.J., De Billy E. (2018). HSF1 Is Essential for Myeloma Cell Survival and A Promising Therapeutic Target. Clin. Cancer Res..

[122] Zaarur N., Gabai V.L., Porco J.A., Calderwood S., Sherman M.Y. (2006). Targeting heat shock response to sensitize cancer cells to proteasome and Hsp90 inhibitors. Cancer Res..

[123] Yang W., Cui M., Lee J., Gong W., Wang S., Fu J., Wu G., Yan K. (2016). Heat shock protein inhibitor, quercetin, as a novel adjuvant agent to improve radiofrequency ablation-induced tumor destruction and its molecular mechanism. Chin. J. Cancer Res..

[124] Iwasaki S., Floor S.N., Ingolia N.T. (2016). Rocaglates convert DEAD-box protein eIF4A into a sequence-selective translational repressor. Nature.

[125] Chen R., Wierda W.G., Chubb S., Hawtin R.E., Fox J.A., Keating M.J., Gandhi V., Plunkett W. (2009). Mechanism of action of SNS-032, a novel cyclin-dependent kinase inhibitor, in chronic lymphocytic leukemia. Blood.

[126] Wu Y., Chen C., Sun X., Shi X., Jin B., Ding K., Yeung S.C., Pan J. (2012). Cyclin-dependent kinase 7/9 inhibitor SNS-032 abrogates FIP1-like-1 platelet-derived growth factor receptor alpha and bcr-abl oncogene addiction in malignant hematologic cells. Clin. Cancer Res..

[127] Rye C.S., Chessum N.E., Lamont S., Pike K.G., Faulder P., Demeritt J., Kemmitt P., Tucker J., Zani L., Cheeseman M.D. (2016). Discovery of 4,6-disubstituted pyrimidines as potent inhibitors of the heat shock factor 1 (HSF1) stress pathway and CDK9. Medchemcomm.

[128] Ganguly S., Home T., McGuirk J., Rao R., Kambhampati A.Y., Shi H., Dandawate P., Saluja S.P., McGuirk J., Rao R. (2015). Targeting HSF1 disrupts HSP90 chaperone function in chronic lymphocytic leukemia. Oncotarget.

[129] Sangwan V., Banerjee S., Jensen K.M., Chen Z., Chugh R., Dudeja V., Vickers S.M., Saluja A.K. (2015). Primary and liver metastasis-derived cell lines from KrasG12D; Trp53R172H; Pdx-1 Cre animals undergo apopto-sis in response to triptolide. Pancreas.

[130] Heimberger T., Andrulis M., Riedel S., Stuhmer T., Schraud H., Beilhack A., Bumm T., Bogen B., Einsele H., Bargou R.C. (2013). The heat shock transcription factor 1 as a potential new therapeutic target in multiple myeloma. Br. J. Haematol..

[131] Yoon T., Kang G.Y., Han A.R., Seo E.K., Lee Y.S. (2014). 2,4-Bis(4-hydroxybenzyl)phenol inhibits heat shock transcription factor 1 and sensitizes lung cancer cells to conventional anticancer modalities. J. Nat. Prod..

