# Supplementary material for: Heat Shock Factor 1 Inhibition: A Novel Anti-Cancer Strategy with Promise for Precision Oncology
Source: Cancers (Basel). 2023 Oct 27;15(21):5167. doi: 10.3390/cancers15215167 (PMC10649344; doi:10.3390/cancers15215167)
Supplement: Supplementary file 1 [file cancers-15-05167-s001.zip › cancers-2652374-SI.pdf]

Supplementary File

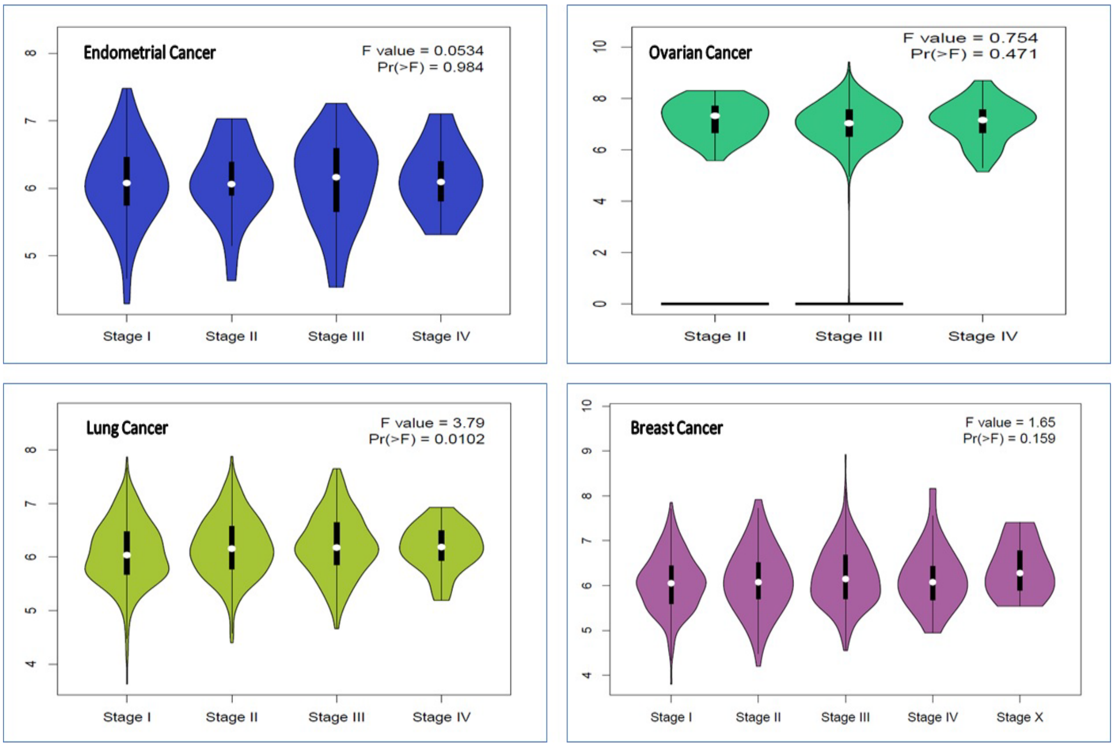

S1. The association of HSF1 expression with clinical stages. The analysis shows that lung cancer showed statistically significant differences in HSF1 expression at different stages, but there are no statistically differences in the other three cancer types we analysed. The data was retrieved and analysed from http://gepia2.cancer-pku.cn/#analysis

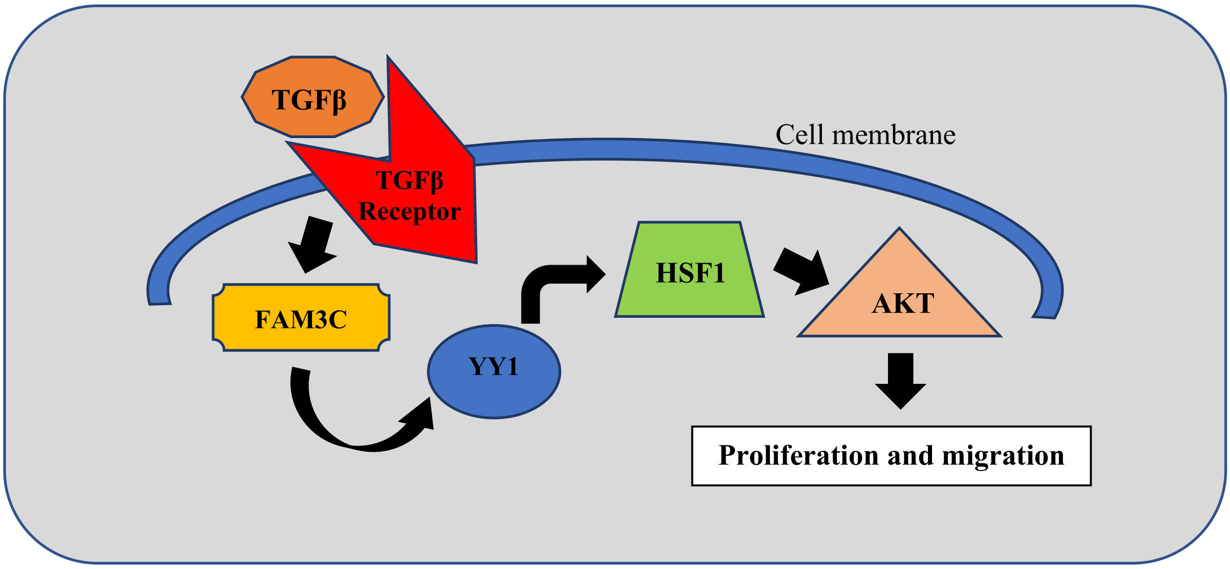

S2. FAM3C-YY1-HSF1 signalling axis in the pathogenesis of breast cancer. FAM3C-YY1-HSF1 signalling axis is essential for TGFβ-promoted proliferation and migration of breast cancer cells.

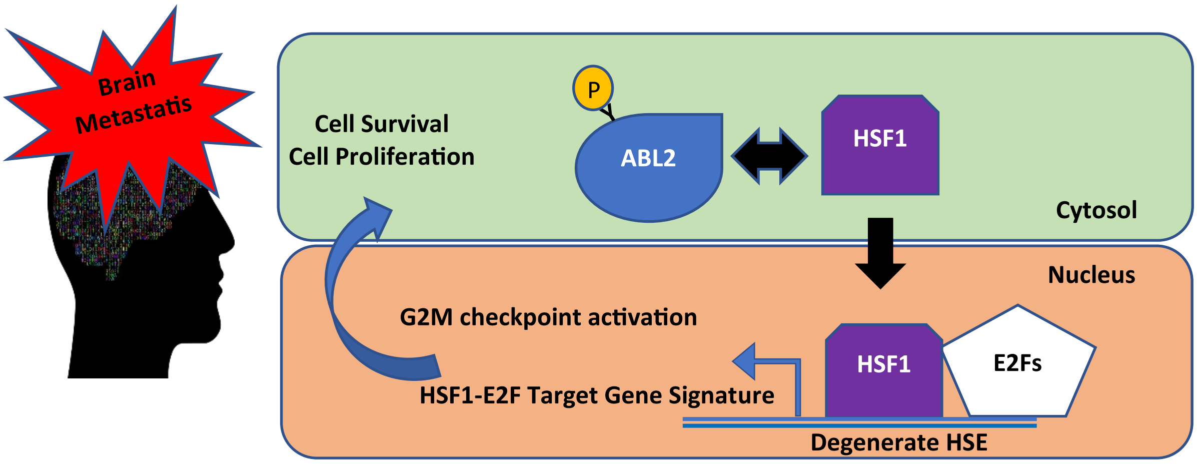

S3. Model diagram illustrating ABL2-HSF1-E2F signaling in lung adenocarcinoma brain metastasis. First, ABL2 interacts with HSF1. HSF1 will oligomerize and accumulate in the nucleus in response to stress by binding to degenerate HSE of E2F target genes. The result of this process is the activation of the G2/M checkpoint which contributes to cell survival and cell proliferation.

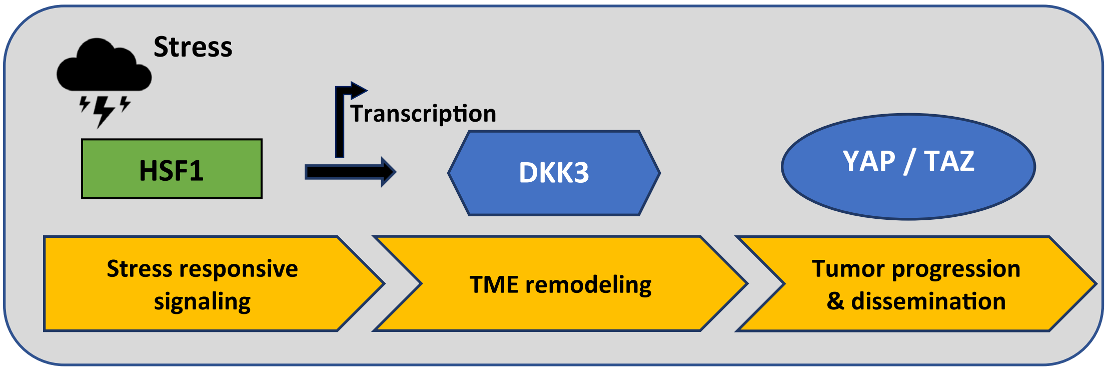

S4. HSF1 in CAFs upregulates DKK3 which potentiates YAP/TAZ signaling. YAP promotes actomyosin contractility leading to extracellular matrix (ECM) remodeling and cancer cell growth and invasion.
